# A New Zealand regional work-related sprains and strains surveillance, management and prevention programme: study protocol

**DOI:** 10.1186/s12891-022-06094-y

**Published:** 2022-12-31

**Authors:** Ian Laird, Justine McIntyre, Barry Borman, Rod Adank, Liz Ashby

**Affiliations:** 1 School of Health Sciences, College of Health, Massey University, Palmerston North, New Zealand; 2 Hastings Health Centre, Hastings, New Zealand; 3 Research Centre for Hauora and Health, College of Health, Massey University, Wellington, New Zealand; 4 School of Design, College of Creative Arts, Massey University, Wellington, New Zealand

**Keywords:** Sprains and strains, Surveillance, Prevention through design, Intervention, Agriculture, Horticulture

## Abstract

**Background:**

The impact and costs associated with work-related sprains and strains in New Zealand and globally are substantial and a major occupational and public health burden. In New Zealand around one-third of all sprains and strains workers compensation (ACC) claims (2019) are for back injuries, but shoulder and arm injuries are increasing at a faster rate than other sprain and strain injuries (ACC, 2020). A need exists for a change to current approaches to sprains and strains prevention, to more effectively manage this significant and persistent problem in workplaces. Designing out hazards is one of the most effective means of preventing occupational injuries and illnesses. This paper outlines the study protocol of the surveillance, management and prevention programme and describes the utilisation of prevention through design principles in the prevention of work-related sprains and strains in agriculture/horticulture/food production in the Hawkes Bay region of New Zealand.

**Methods:**

This is a prospective mixed methods study incorporating the collection of quantitative data to describe the epidemiology of work related sprains and strains injuries presenting to the regional health centre (Hastings Health Centre) over a period of 24 months and qualitative data from participants presenting at the health centre to identify high risk industry sectors/ occupations/ workplaces and tasks and design, develop and apply prevention through design principles/ solutions/interventions to critical features of the work and work environment and undertake an outcome evaluation during the last 6 months of the project.

**Discussion:**

The purpose of this project is to establish an epidemiological surveillance programme to assess the incidence and prevalence of work-related sprains and strains according to age, sex, industry sector and occupation to target efforts to prevent work-related sprains and strains, by applying prevention through design (PtD) principles in selected workplaces in agriculture. The collection of more detailed case, occupational and work history data from a sample of patients presenting at the HHC clinic will identify high risk industry sectors/occupations/workplaces and tasks. Assessment techniques will include comprehensive design, design thinking and human factors/ergonomics methodologies through co-design and participatory ergonomics techniques. The PtD solutions/ interventions implemented will be evaluated using a quasi-experimental design consisting of a pre-test/ post-test with-in subjects design with control groups that do not receive the intervention.

**Supplementary Information:**

The online version contains supplementary material available at 10.1186/s12891-022-06094-y.

## Background

Sprains and strains are an umbrella term that refer to damage to the soft tissues in the body, including ligaments, tendons, and muscles. They are common injuries that share some symptoms but affect different body parts. The impact and costs associated with work-related sprains and strains in New Zealand are substantial and a major public and occupational health burden. Over the past 5 years, New Zealand workers compensation sprains and strains claims have increased significantly compared with other types of injuries. In 2017, sprains and strains made-up the highest proportion of work-related claims (38%), and these claims amount to the highest level of Accident Compensation Corporation (ACC) expenditure on work-related injuries (41%). These claims cost NZ$132 million (2017) and resulted in at least 1.3 million lost workdays. Around one-third of all sprains and strains claims (2019) are for back injuries, but shoulder and arm injuries are increasing at a faster rate than other sprain and strain injuries. Injuries to the back and upper body are the most likely body sites to become higher cost [1].

Most current interventions for sprains and strains prevention are focused on changing an individual’s behaviour, reducing task specific hazards, work organisation and workplace environment factors with little consideration of the broader contextual factors which are associated with the complex aetiology of sprains and strains. Despite the apparent success of some multiple component interventions, the inability to identify which elements of the intervention were effective makes replication problematic. Significant changes in injury prevention practice are required to address this problem, including a shift in emphasis from strategies aimed at changing behaviour, to more comprehensive approaches which take into account all aspects of an individual’s work, and which also draw upon the subject matter expertise of those doing the work and incorporate the wider context in which the work is taking place.

A need exists for a change to current approaches to sprains and strains prevention, to more effectively manage this significant and persistent problem in New Zealand workplaces. Designing out hazards is one of the most effective means of preventing occupational injuries and illnesses. Prevention through Design (PtD), a systems-based framework developed by NIOSH in 2007, extends the traditional hierarchy of controls by focusing on hazard elimination, followed by risk minimization through the application of design, redesign, and retrofit activities. Although this concept is well known, there has not been a concerted effort to achieve broad implementation of it, particularly in relation to sprains and strains. The PtD approach consists of developing collaborations or partnerships, procedures, resources, implementation plans, design strategies, case studies, and research to practice initiatives from identification of the problem to implementation. Applying an intervention and design thinking approach to sprains and strains prevention is innovative and complements other traditional approaches. In addition, the benefit of PtD is that innovation and productivity are key considerations at the outset, and also employee ‘buy in’ is optimised, and productivity enhanced or improved.

There are numerous examples of successful prevention efforts through design in the literature [2–7]. Unfortunately, there are few universal design interventions aimed at prevention because of the fact that all job tasks are different [8]; however, those that are similar in nature may benefit from cross-job task transfer of design principles. Whenever possible, past preventive solutions to similar job tasks should be sought out and improved upon (if possible) for use in the design of future job tasks. If no similar situations are found, a comprehensive ergonomic examination will be necessary. The design solutions identified from the detailed ergonomics evaluations should then be implemented in conjunction with multicomponent programmes with facets of psychosocial and organizational work components in order to enhance the likelihood of success as a means of work-related musculoskeletal disorder elimination.

The aim of this paper is to outline the general design and study protocol of the surveillance, management and prevention programme and to describe the type of information that each of its components can provide.

## Methods/design

### Aim

The Hawkes Bay Sprains and Strains Surveillance, Management and Prevention Programme is aimed at estimating the nature and extent of work-related sprains and strains injuries (WMSD’s) in the agricultural/horticulture/food production sectors in the Hawkes Bay region of the North Island of New Zealand and assessing workplaces to identify where there are opportunities of designing out the cause of these injuries utilising prevention through design (PtD) principles.

### Objectives

The objectives of this project will be to:

- Objective 1: Identify and engage with key community, stakeholder and industry groups critical to sprains and strains prevention in the Hawkes Bay region within the first 6 months of the project and develop partnership agreements.
- Objective 2: Establish an epidemiological surveillance programme for sprains and strains (WMSD) in the Hawkes Bay region based at the Hastings Health Centre within the first 6 months of the project.
- Objective 3: Collect case, occupational and work history data from sprains and strains patients within the Hawkes Bay population from 2022 to 2024 (Years 1, 2 and 3) and analyse data to identify high risk industry sectors/occupations/workplaces and tasks.
- Objective 4 – Assess those high-risk industry sector workplaces/tasks (identified in Objective 3) for the primary prevention of sprains and strains (Year 1) and design and apply prevention through design principles/ solutions/interventions to critical features of work that are related to incidence of sprains and strains in Year 2 of the project.
- Objective 5: Undertake an evaluation of the PtD solutions/interventions implemented in Year 3 of the project.

### Research design

This is a prospective mixed methods study incorporating the collection of quantitative data to describe the epidemiology of work related sprains and strains injuries presenting to the regional health centre (Hastings Health Centre, (HHC)) over a period of 24 months and qualitative data from participants presenting at the health centre to identify high risk industry sectors/ occupations/ workplaces and tasks and design, develop and apply prevention through design principles/ solutions/interventions to critical features of the work and work environment and undertake an outcome evaluation during the last 6 months of the project.

The study has two design components. (1) An observational (cross-sectional) surveillance study to assess the incidence and prevalence rates of work-related sprains and strains in the regional (Hawkes Bay) workforce according to sex, ethnicity, age group, industry sector, occupation, injury diagnosis, injury site, injury cause; and sample case, occupational and work history survey for patients presenting to the health centre with work-related sprains and strains from 2022 to 2024 (Years 1, 2 and 3) will be collected, and (2) a quasi-experimental intervention study, where the solutions/ interventions implemented will be evaluated using a quasi-experimental design consisting of a pre-test/ post-test with-in subjects design with control groups that do not receive the intervention. The evaluation will be undertaken in Years 2 and 3 of the project (2023/2024).

### Inclusion and exclusion

A cohort of eligible patients will be recruited by the project through collaboration with the clinical team at the HHC. This cohort will consist of patients who have presented with relevant injuries (upper extremity located sprains & strains). They will have agreed to provide additional case information, along with occupational work history data. Eligible patients will be above the age of 18 years.

#### Inclusion criteria

- Patients who visit their general practitioner/clinic with a new complaint or new episode of complaint of the neck, shoulder, elbow, wrist, hand, arm.
- Injury is regarded as work-related.
- 18 years or above and capable of filling in the questionnaires.
- Patients can be included in the study only once.
- An episode of complaint is considered ‘new’ if patients have not visited their GP for the same complaint during the preceding 3 months.

#### Exclusion criteria

- Repeated attendance in the same (urgent care) facility for the same injury.
- Patients are excluded from the study if a malignancy, prosthesis, amputation or congenital defect causes the presented complaint.

The scope of the project is associated with upper extremity located sprains and strains. Consenting adults over the age of 18 will be an important factor in order to obtain the necessary permissions directly from the patient concerning their involvement in the study.

Beyond these factors, the study will be extremely inclusive with the involvement of patients across all genders, age groups, cultures and sociodemographic groups. A large proportion of agricultural workers within the Hawke’s Bay region are of Māori/Pasifika background. We therefore consider it likely that our participating cohort of patients will represent this also.

### Study setting

Hawke’s Bay is a region on the east coast of New Zealand’s North Island. The region is renowned for agricultural production including horticulture, with large orchards and vineyards on the plains. In the hilly parts of the region sheep and cattle farming predominates, with forestry blocks in the roughest areas. Hawke’s Bay has 17,886 ha (44,200 acres) of horticultural land, the third largest area in New Zealand. The largest crops by land area are apples (4750 ha), wine grapes (3620 ha), squash (3390 ha), and peas and beans (1360 ha).

Population growth is an indicator of a region’s attractiveness as a place to live and work. A strong regional economy with plentiful job opportunities will help a region retain its population and attract new residents from other regions and abroad. This section contrasts Hawke’s Bay Region’s recent population growth with other districts and the country as a whole. Hawke’s Bay Region’s population was 178,600 in 2020, up 2.0% from a year earlier. Based on the profile of Hawkes Bay industry in 2019, agriculture accounts for 15% of all employed in the region.

The Hawkes Bay region had the highest incidence rate of work-related compensation claims in 2019, with 146 claims per 1000 FTEs.

### Sample size

Surveillance study: Patients presenting at the Hastings Health Centre, with a new work-related sprain and strain injury of any kind will be included in the surveillance study. Data will be obtained from patients presenting with injuries between 1st of June 2022 and 31st March 2024 inclusive. Sample size estimation will not be required, as this is a prospective observational study of all cases presenting with injuries who meet the inclusion criteria. Data from the health centre patient management system for 2020/2021 estimated there may be 20 patients per week presenting with work-related sprain and strain injuries that are eligible for workers compensation. The study team will expect to see around 5 injuries per day and about 3000 injury cases over the course of 24 months.

Case, occupational and work history study: The sample size for an annual population of 1500 patients presenting at the health centre, 95% CL, margin of error 5% is 300 participants. This indicates a sampling frequency of 6 participants a week.

### Data collection

#### Surveillance data

Epidemiological surveillance data will be based on physician/surgeon case-reports using the Medtech-32 patient management system (PMS) (Medtech Global Ltd) at the Hastings Health Centre and ACC claims data will be used to document patterns of work-related sprains and strains in the Hawkes Bay region from September 2021 to December 2023. The epidemiological surveillance programme will be a separate project database with de-identified and anonymised case information that will be linked to the Medtech system.

The surveillance system will support the identification of primary trends, which will in turn enable the project to deliver specific and targeted efforts to prevent workplace-initiated sprains and strains. Summarised anonymised/de-identified data will be collected and presented on a quarterly basis. The data reported will be comprised of the following data categories; Sex, Ethnicity, Age Group, Industry Sector, Occupation, Injury Diagnosis – (Sprains/Strains/Soft Tissue Injury (including gradual process injuries (carpel tunnel syndrome)), Injury Site (Upper Extremity) and Injury Cause.

**Fig. 1:**
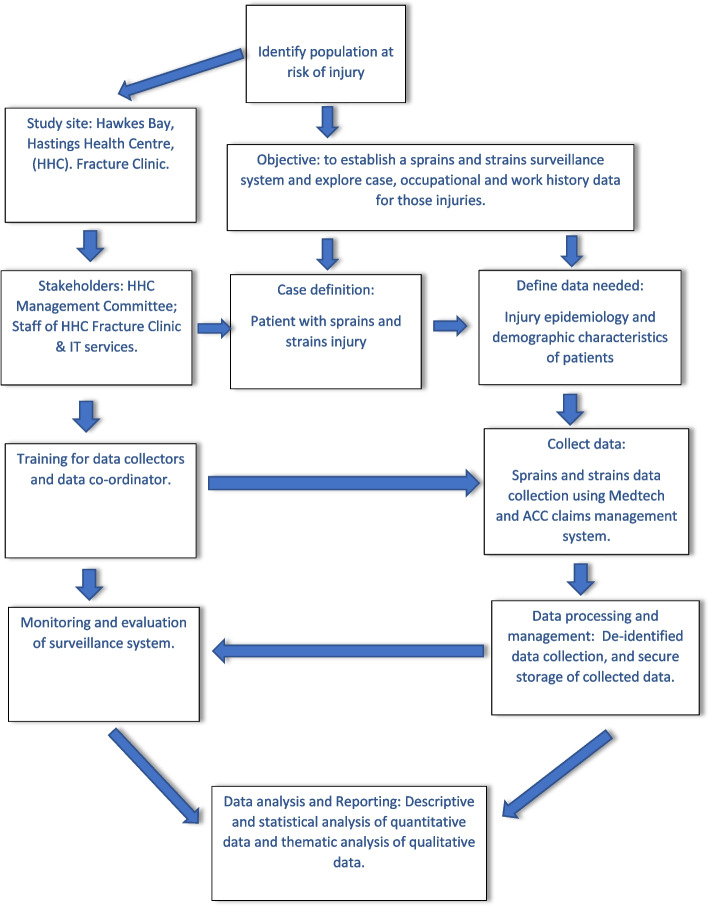
Conceptual framework for the Hawkes Bay sprains and strains surveillance system

The conceptual framework for data collection for the surveillance system is presented in Fig. 1.

#### Case, occupational and work history data

Once the clinical consultation has been given, patients presenting at the Fracture Clinic, Hastings Health Centre will be approached to solicit interest and consent for participation in the research. Case, occupational and work history data from patients presenting with sprains and strains within the Hawkes Bay population from 2022 to 2024 (Years 1, 2 and 3) will be collected. Standardised MSS (Nordic Musculoskeletal Questionnaire) and PSC-12, occupational and work history survey questionnaires will be utilized (Participant Questionnaire). Data will be analysed to identify high-risk (work-related sprains and strains) industry sectors within agriculture and related occupations/ workplaces/ tasks. De-identified and anonymised case information will be recorded.

In addition, workplace assessments will be undertaken with industrial partners, to identify the incidence of sprains and strains cases which may not seek medical attention, and therefore will not be included in data from the HHC surveillance system.

#### Pretesting and training

1. Epidemiological surveillance system. Pilot data from a sample of the Medtech-32 patient management system (Medtech™) was assessed to determine its adequacy/appropriateness for epidemiological surveillance.
2. The occupational, case and work history data collection questionnaire was pretested prior to survey commencement. Minor revisions to the questionnaire fields were made as a consequence of the pre-test, before formal data collection commenced.

Training of research team personnel collecting the data was undertaken, prior to survey commencement, to ensure consistency of data collection protocols and interview techniques.

A training workshop was provided for data collectors that included background information on collection of data for the project; the inclusion/exclusion criteria; detailed overview of the questionnaire and how information should be recorded; and ethical issues relating to the collection of data and culturally appropriate protocols for interviewing Māori and Pacific Island participants.

Data collectors were followed up after the training, to ensure they were comfortable with completion of the sections in the questionnaire and identifying any issues participants had in completing the questionnaire. The opportunity to provide feedback comments to the project team was important during the pilot phase, and minor modifications to the data collection process and language were made.

#### Quality assurance

The following mechanisms will be used to ensure adequate data quality:

1. Case ascertainment: The Quality Assurance Specialist will examine the PMS data for missing or incorrectly coded patient data through the Clinical Audit Tool of Medtech. The data will be monitored weekly and a regular report (monthly) of the number of missing cases will be recorded.
2. Data validation: The assessment will involve sampled, double data entry data for the PMS data every 8 weeks. Two data collectors will each complete a surveillance form for the same patient with subsequent comparison by the data supervisor. The outcome will be a measure of the levels of agreement between the two data collectors.

#### Qualitative data collection

A cohort of eligible patients will be recruited by the project through collaboration with the clinical team at the Hastings Health Centre. This cohort will consist of patients who have presented with relevant injuries (upper extremity located sprains & strains). They will have agreed to provide additional case information, along with occupational work history data. This data will be collected via our Occupational History Questionnaire. This consists of a standardised Nordic Musculoskeletal Questionnaire (NMQ), a Psychological Safety Climate (PSC) – 12 Point Survey, as well as an individual occupational history questionnaire. The Occupational History Questionnaire will be made available in both printed and electronic versions.

### Data management and analysis

A Data Management Plan has been developed for the study, involving data collection, analyse and storage of data from the surveillance system and the case, occupational and work history survey.

#### Surveillance data

For the surveillance system patient data, the PMS is confidential and only clinic clinical staff are able to access files, in addition, only deidentified summarised data will be analysed and reported on. The final nonidentifiable, encrypted dataset will be exported from Medtech-32 and all injury data collected during the study period will be analysed. Descriptive and analytical analysis will be performed using SPSS [9]. Analysis will be conducted to describe patterns and outcomes of injury using basic frequencies, 95% confidence intervals, means and standard deviations. Where feasible and appropriate, associations between socio-demographics, patterns of injury and severity will be explored using multivariable regression analysis, reporting through odds ratios (OR), *P* value (0.05 significant level) and confidence intervals (CI 95%).

#### Qualitative data

The case, occupational and work history data in de-identified form will be stored locally and allocated a unique study identifier to enable linkage to the PMS. All participants will be allocated a unique study identifier and all patient data will be de-identified. Research project personnel will not have access to the PMS where the health data is stored. There will be no possibility of re-identification. Thematic analyses will be undertaken on the data form the standardised Nordic Musculoskeletal Questionnaire (NMQ), and the Psychological Safety Climate (PSC) – 12, as well as an individual occupational history data. Descriptive summarised data will be reported on.

### Ethical considerations

All participants will be informed of, and provide consent for, the collection and use of their data for the purposes of this study, and for any mandatory secondary uses. Eligible participants will be screened by clinical staff within the Fracture Clinic at HHC. Staff will clearly indicate that the research is currently being undertaken, and that their personal details used in the Patient Management System will not be used in the research (deidentified). Only summarised data will be reported.

Following their consultation, and upon approval to provide further information to the project, participants will be directed to research team personnel to conduct the detailed survey. Data will be collected either in written form (via interview) or electronically (iPad or similar).

Given the nature of the employee demographic across our target sectors, there is a high likelihood that we will be engaging with patients for whom English is not a written/spoken language. The project will therefore seek to ensure that documentation is available in multiple languages, including Māori, Samoan, Fijian and Tongan.

The study requires a single interview during which the Occupational & Work History Questionnaire will be completed. This is expected to take approximately 20 minutes per participant. This will be the only time commitment required. If the participant agrees to participate in the study, they will be provided with a Participant Information Sheet (PIS) which will be explained and a Participant Informed Consent Form (PICF), to complete. The Occupational & Work History Questionnaire will then be completed either (a) at the Clinic in the Hastings Health Centre, or (b) through an electronic questionnaire, where the participants are provided with an internet link to access the questionnaire.

The Participant Information Sheet and Informed Consent form has been pre-tested with workers who volunteered to complete a readability test of the documents. In addition, an online Text Readability Consensus Calculator which uses readability formulas to calculate the average grade level, reading age, and text difficulty of sample text. Results indicated an Flesch Reading Ease score of 67 for the sample text, equivalent to standard, average reading score (8th grade).

The data collection will be consistent with the current national COVID-19 regulations (https://covid19.govt.nz/) and current Massey University guidelines (Massey University Operating Plan for the COVID-19 Protection Framework).

The project and study protocol have been reviewed and approved by the Massey University Human Ethics Committee: Southern A, Application 22/06. Ethical aspects of the application were also considered and approved by the Northern A Health and Disability Ethics Committee (HDEC, Ministry of Health) (Ethics Ref: 2022 EXP 12321).

## Discussion

We believe this is the first attempt to establish, design and implement an epidemiological surveillance system for the surveillance of work-related sprains and strains in New Zealand. In addition, the study will effectively utilise the injury surveillance data in the identification of at-risk agricultural industry sectors and workplaces and assess those high-risk industry sector workplaces/tasks identified for the primary prevention of work-related sprains and strains. Assessment techniques will include comprehensive design, design thinking and human factors/ergonomics methodologies. Workers will be engaged through co-design and participatory ergonomics techniques.

Utilising the design thinking framework, the prevention through design (PtD) process involves: (1) identifying the issues related to sprains and strains and (2) understanding the issues through gathering information and identifying hazards/risk of work-related sprains and strains (3) developing design options through analysis and evaluation of the risks of work-related sprains and strains and generating multiple solutions to eliminate those hazards/risks, (4) identifying the preferred options and selecting an appropriate solution to eliminate the risk of work-related sprains and strains.

Working with regional workers representatives and associations, a participatory ergonomics approach is a key avenue for consulting and engaging with workers in the agricultural sector. Education and promotion of PtD principles for representative groups within the key industry sectors will be developed and provided.

Implementation of the PtD interventions involves the development of prototypes or work design changes and applying those PtD solutions/ interventions in the workplaces, tasks, operations, environments identified. The identified preferred options will aim to be low costs/high effectiveness interventions. The implementation phase will be in year 2 of the project (2022/2023).

The PtD solutions/ interventions implemented will be evaluated using a quasi-experimental design consisting of a pre-test/ posttest with-in subjects design with control groups that do not receive the intervention. Quasi-experimental designs allow for a means of compromise between the practical workplace restrictions and the rigor required for demonstrating intervention effectiveness. Quasi-experimental designs are structured similar to experimental designs, but the participants and control group are created through a non-random process [8]. The evaluation will be undertaken in Years 2 and 3 of the project (2022/2023).

The evaluation of the project will be modelled on the Health Impact Assessment model developed by and promoted by the New Zealand Ministry of Health [10]. The evaluation plan will involve process, impact and outcome evaluation methodologies. Both qualitative and quantitative data are used in the evaluation of then project. The project evaluation will be undertaken in Years 2 and 3 of the project (2023/2024).

The anticipated outcomes and benefits of the project will be: (1) Sustained collaboration between industry leaders, businesses, local iwi, unions, workers, health care providers and government agencies in the Hawkes Bay to drive initiatives which reduce the risk of workplace related sprains and strains in the agriculture sector. (2) A measurable reduction in the number of work-related sprains and strains claims from the target sector and improved psychosocial risk management. (3) Principles of the Prevention through Design approach are increasingly implemented across target sector workplaces, within 5 years.

The primary limitation of the study is use of the Hastings Health Centre data as an injury surveillance system. There is potential for selection bias because it will only identify people with injuries presenting to the health centre and will therefore not include patients seeking care from other community-based health services in the Hawkes Bay or providing self-care. The study will therefore underestimate the true burden of sprains and strains injuries in the Hawkes Bay communities being studied. This is an important consideration when interpreting the data and generalising the findings beyond the region.

A strength of the study design is that this study will collect all injury cases reported over a period of 24 months and will therefore not be subject to seasonal variations in injury incidence (for example fruit picking, harvesting and processing). Another strength of the study is the utilisation of the surveillance system in the evaluation of the outcomes of the prevention through design interventions/solutions implemented in the later stages of the project.

This study seeks to establish an injury surveillance system to determine the nature, extent and burden of work related sprains and strains injuries affecting agricultural/horticultural/meat processing workers in the Hawkes Bay region of New Zealand. In addition, it will design, develop, test and apply prevention through designs interventions/solutions to identified at risk work environments, processes and tasks. The objective of which is to have a sustained reduction in work-related sprains and strains incidence beyond the duration of the project, and potentially an opportunity to scale-up the interventions/solutions to the agricultural/horticultural industry sector nationally.

## Supplementary Information

**Additional file 1.** Participant Questionnaire.

## Data Availability

The datasets that are going to be generated and analysed during the current study will not be made publicly available due to ethics protocols and national regulations.

## Abbreviations

ACC: Accident Compensation Corporation, National workers compensation insurance scheme
CL: Confidence Limit
HHC: Hastings Health Centre, location of study in Hawkes Bay region
NMQ: Nordic Musculoskeletal Questionnaire
PIS: Patient Information Sheet
PICF: Patient Informed Consent Form
PMS: Patient Management System
PtD: Prevention through Design
PSC-12: Psychological Safety Climate-12 questionnaire
Standardised MSS: Standardised Musculoskeletal Symptoms
