# Supplementary material for: A New Zealand regional work-related sprains and strains surveillance, management and prevention programme: study protocol
Source: BMC Musculoskelet Disord. 2022 Dec 31;23:1143. doi: 10.1186/s12891-022-06094-y (PMC9803593; doi:10.1186/s12891-022-06094-y)
Supplement: Supplementary file 1 — Additional file 1. Participant Questionnaire. [file 12891_2022_6094_MOESM1_ESM.pdf]

The Hawkes Bay Sprains and Strains Surveillance, Management and Prevention Programme

Occupational History Questionnaire

Subject ID #: ☐☐☐☐☐☐

Today's date: ☐ Day ☐ Month ☐ Year

Participant Age Group: ☐ 18-24 Years ☐ 25-34 Years ☐ 35-44 Years ☐ 45-54 Years ☐ 55-64 Years ☐ 65 + Years

Gender: ☐ Male ☐ Female ☐ Prefer Not to Say ☐ Other

Specify: ______

To which ethnic group (or groups) do you belong?

☐ European/NZ ☐ Maori ☐ Pacific Island ☐ Other

Specify: ______

Part 1: Lifetime Work History

1. Please tell me all the jobs you have held in order from the first job you ever held to the most recent job ever held.

Interviewer:
Please include all jobs that lasted at least 6 months in total. Please start with the first job after leaving school and end with the most recent.
The list should be without gaps, meaning that also e.g. unemployed periods or periods taking care of children should be reported here.
The last year in the work history should be the year of interview.

**Job Number**	Employment Sector? (Sector and Region)	Over what **period** did you work for this employer?	What was the **main activity** of the **company** or **organisation** you worked for? *(For example: sheep farming, selling shoes, making clothes)*	What was your job **function/role**?
**1.**	Sector …………………<br>Geographical Region …………………	From: …….. *(year)*<br>To: …….. *(year)*<br>Total time employed: …………... *years*		Job title: ……………….<br>Prefer not to Say ☐
**2.**	Sector …………………<br>Geographical Region …………………	From: …….. *(year)*<br>To: …….. *(year)*<br>Total time employed: …………... *years*		Job title: ……………….<br>Prefer not to Say ☐
**3.**	Sector …………………<br>Geographical Region …………………	From: …….. *(year)*<br>To: …….. *(year)*<br>Total time employed: …………... *years*		Job title: ……………….<br>Prefer not to Say ☐

	**4.**	Sector<br>…………………<br>Geographical Region<br>…………………	From:<br>…….. *(year)*<br>To:<br>…….. *(year)*<br>Total time employed:<br>………….. *years*		Job title:<br>……………….<br>Prefer not to Say<br>☐
	**5.**	Sector<br>…………………<br>Geographical Region<br>…………………	From:<br>…….. *(year)*<br>To:<br>…….. *(year)*<br>Total time employed:<br>………….. *years*		Job title:<br>……………….<br>Prefer not to Say<br>☐
	**6.**	Sector<br>…………………<br>Geographical Region<br>…………………	From:<br>…….. *(year)*<br>To:<br>…….. *(year)*<br>Total time employed:<br>………….. *years*		Job title:<br>……………….<br>Prefer not to Say<br>☐
	**7.**	Sector<br>…………………<br>Geographical Region<br>…………………	From:<br>…….. *(year)*<br>To:<br>…….. *(year)*<br>Total time employed:<br>………….. *years*		Job title:<br>……………….<br>Prefer not to Say<br>☐
	**8.**	Sector<br>…………………<br>Geographical Region<br>…………………	From:<br>…….. *(year)*<br>To:<br>…….. *(year)*<br>Total time employed:<br>………….. *years*		Job title:<br>……………….<br>Prefer not to Say<br>☐

Part 2: Your current or most recent job

I will now ask you some more questions about **your current job.**

Interviewer: *Here we include only jobs for pay, profit or income.*
This part needs to be completed for all the jobs the respondent is currently holding. For example, if someone worked days in a factory and evenings as a cleaner, part 2 needs to be completed twice (use add-in). If the subject has no current job (e.g. is unemployed, taking care of children, sick leave, retired): this part should be completed for the last job held

Period of Employment:

1. Started ……………… *(year)* To ………………… *(year)*

Interviewer: *if the subject still works in this job, please write down CURRENT for end-year*

2. How many **hours per week** do you work in this job? (on average)……………… *(hours per week)*

3. How many **days per week** do you work in this job? (on (on average):………………….. *(days per week)*

4. Do you regularly work **outside 8-5pm** for this job?

YES NO

if yes, please specify:
……. ……………………………….
……...………………………………
……………………………………
……………………………………

5. What is the **main activity** of the company or organisation you work for?

(for example: what was produced, what service was provided)

…………………………………………………
…………………………………………………
…………………………………………………
…………………………………………………
…………………………………………………
…………………………………………………

6. What **department** do you work in?

…………………………………………………

7. What is **your job title?**

…………………………………………………

8. Please **describe your specific job** in detail:

What do you do?

How do you do it?

What **materials** do you use?

What **tools** or **machinery** do you use?

What type of **process** is it?

Interviewer: Try to go through each point e.g: what do you do, how do you do it etc. If no response: ask respondent to describe a typical working day.

8.(Cont.)

The Hawkes Bay Sprains and Strains Surveillance, Management and Prevention Programme

Part 3: MSD & Employee Questionnaire

We would like you to complete this questionnaire which is interested in you and the work you perform. This forms part of our study which is looking at work related sprains and strains. Please try to answer all of the questions. The information you provide is strictly confidential.

1 Job Type / Key tasks / Activities

2 Are you right or left handed? *Right handed* ☐ *Left handed* ☐

3 Do you rotate or change what tasks you do during each shift *Yes* ☐ *No* ☐

(a) If YES, on average how often

1 ☐ *Every hour*
2 ☐ *Once every 2 hours*
3 ☐ *Once every 2-4 hours*
4 ☐ *More than 4 hours*

(b) Does your supervisor encourage/enforce your rotation? *Yes* ☐ *No* ☐

4 Do you work in any other jobs as well as this one? *Yes* ☐ *No* ☐

5 If YES how many hours per week do you work in other jobs? ☐ *hrs*

6 What other type of jobs do you do? ☐

ABOUT YOUR HEALTH

7 Have you had any muscular aches, pains or discomfort during the last **12 months**? *Yes* ☐ *No* ☐
Do not include pain associated with menstrual periods or pregnancy

8 Have you had any muscular aches, pains or discomfort during the last **7 days**? *Yes* ☐ *No* ☐
Do not include pain associated with menstrual periods or pregnancy

If **No** to **BOTH** 7 and 8 stop here

9 Please place a tick in the box where you have had muscular aches, pains or discomfort, using the picture as a guide.

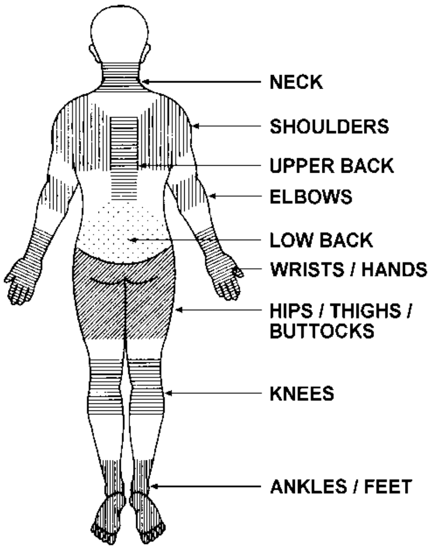

	Pain in the last *7 days*	*Pain in the last* *12 months*	*In the last **12 months**, has pain in this area stopped you doing normal activities e.g. hobbies, housework*
Neck			
Right shoulder			
Left shoulder			
Right elbow			
Left elbow			
Right wrist & hand			
Left wrist & hand			
Upper back			

10 Have you ever taken advice for these aches and pains *e.g. seen the occupational health nurse, or a doctor, osteopath, physiotherapist or similar* *Yes* ☐ *No* ☐

11 Have you had a medical diagnosis from a doctor for these aches and pains? *Yes* ☐ *No* ☐

If YES, please write down the condition

12 In the last 12 months, have you had time off work because of these aches and pains? *Yes* ☐ *No* ☐

(a) If **YES**, how many **times** have you been away from work during during the last 12 months? ☐ *times*

(b) If **YES**, how many **days** have you been away from work in the last 7 days? ☐ *days*

13 Have you changed jobs or duties because of these aches and pains? *Yes* ☐ *No* ☐

If YES, please write down what you have changed

14 Do you think these aches and pains are related to anything you do or equipment you use at work or elsewhere? *Yes* ☐ *No* ☐

If YES, what?

14. Continued….

Part 4: Psychosocial Safety Climate Questionnaire

The following statements concern the Psychological Health and Safety in your workplace. Please answer with the best option provided:

1	In my workplace senior management acts quickly to correct problems/issues that affect employees' psychological health	Strongly Disagree	Disagree	Neither Agree or Disagree	Agree	Strongly Agree
2	Senior management acts decisively when a concern of an employees' psychological status is raised	Strongly Disagree	Disagree	Neither Agree or Disagree	Agree	Strongly Agree
3	Senior management show support for stress prevention through involvement and commitment	Strongly Disagree	Disagree	Neither Agree or Disagree	Agree	Strongly Agree
4	Psychological well-being of staff is a priority for this organization	Strongly Disagree	Disagree	Neither Agree or Disagree	Agree	Strongly Agree
5	Senior management clearly considers the psychological health of employees to be of great importance	Strongly Disagree	Disagree	Neither Agree or Disagree	Agree	Strongly Agree
6	Senior management considers employee psychological health to be as important as productivity	Strongly Disagree	Disagree	Neither Agree or Disagree	Agree	Strongly Agree
7	There is good communication here about psychological safety issues which affect me	Strongly Disagree	Disagree	Neither Agree or Disagree	Agree	Strongly Agree
8	Information about workplace psychological well-being is always brought to my attention by my manager/supervisor	Strongly Disagree	Disagree	Neither Agree or Disagree	Agree	Strongly Agree
9	My contributions to resolving occupational health and safety concerns in the organization are listened to	Strongly Disagree	Disagree	Neither Agree or Disagree	Agree	Strongly Agree
10	Participation and consultation in psychological health and safety occurs with employees, unions and H&S representatives in my workplace	Strongly Disagree	Disagree	Neither Agree or Disagree	Agree	Strongly Agree
11	Employees are encouraged to become involved in psychological safety and health matters	Strongly Disagree	Disagree	Neither Agree or Disagree	Agree	Strongly Agree
12	In my organization, the prevention of stress involves all levels of the organization	Strongly Disagree	Disagree	Neither Agree or Disagree	Agree	Strongly Agree

Hall, Dollard and Coward, 2010. International Journal of Stress Management 2010, Vol. 17, No. 4, 353–383
